# Management of a Large Abdominal Desmoid Tumor Complicated by Lymphedema of the Lower Limb: A Case Report

**DOI:** 10.7759/cureus.64890

**Published:** 2024-07-19

**Authors:** Youssef Ghaddou, Mohamed Bouziane, Abdellah Nouri, Khalid Sair, Abdelaziz Fadil

**Affiliations:** 1 Surgery, Mohammed VI University of Health Sciences, Cheikh-Khalifa International University Hospital, Casablanca, MAR; 2 Visceral Surgery, Mohammed VI University of Health Sciences, Cheikh-Khalifa International University Hospital, Casablanca, MAR; 3 Surgical Gastroenterology, Mohammed VI University of Health Sciences, Cheikh-Khalifa International University Hospital, Casablanca, MAR

**Keywords:** mesh repair, surgical margins, monobloc resection, lymphedema, desmoid tumor

## Abstract

Desmoid tumors, while generally benign histologically, can exhibit locally aggressive behavior, especially when located in the anterior abdominal wall. This case report explores the management of a rare giant desmoid tumor complicated by concurrent lymphedema, emphasizing the nuances of diagnosis, treatment decisions, and their impact on the patient's quality of life. The patient, a 55-year-old obese individual with a BMI of 47, presented with a 25 cm mass in the right paraumbilical region, alongside significant lymphedema in the right lower limb and associated inguinal lymphadenopathy. Abdominal CT revealed a well-defined soft tissue mass in the right paramedian hypogastric region, suggestive of a desmoid tumor. Surgical intervention involved a monobloc resection of the mass with a 5 cm lateral margin, including the right rectus abdominis muscle and associated aponeuroses, and subsequent reconstruction using a biface intraperitoneal synthetic mesh. Postoperative recovery was marked by the resolution of lymphedema and a return to full function of the affected limb. Histopathological examination confirmed the diagnosis of a desmoid tumor. This case underscores the importance of radical surgical resection with adequate margins and appropriate reconstruction to achieve favorable long-term outcomes. The report provides insights for future research and therapeutic advancements in the management of desmoid tumors.

## Introduction

Desmoid-type fibromatosis is a rare mesenchymal neoplasm characterized by local infiltrative growth. Despite its non-metastasizing nature, significant morbidity arises from local complications, including compressive effects at the site of origin. Moreover, there is a notable propensity for high rates of local recurrence following surgical resection. Desmoid-type fibromatosis typically manifests in diverse anatomical locations, prominently involving the extremities, trunk (encompassing the pelvic and shoulder girdles), and the abdominal cavity [1]. 

The management of these tumors represents a multifaceted and personalized endeavor, typically necessitating a comprehensive integration of surgical, radiotherapeutic, and systemic modalities. Determination of the optimal therapeutic approach hinges upon meticulous evaluation of tumor attributes, anatomical site, and individual patient considerations [2].

This case report explores the management challenges presented by a rare occurrence: a giant desmoid tumor in a single patient complicated by lymphedema in the anterior abdominal wall. This unique clinical scenario sheds light on the complexities of diagnosis, treatment decisions, and their impact on the patient's quality of life. Against the backdrop of evolving understanding of desmoid tumor pathogenesis and their relationship with the lymphatic system, this study examines the manifestations and management intricacies of giant desmoid tumors in this anatomical context. 

## Case presentation

**Figure 1:**
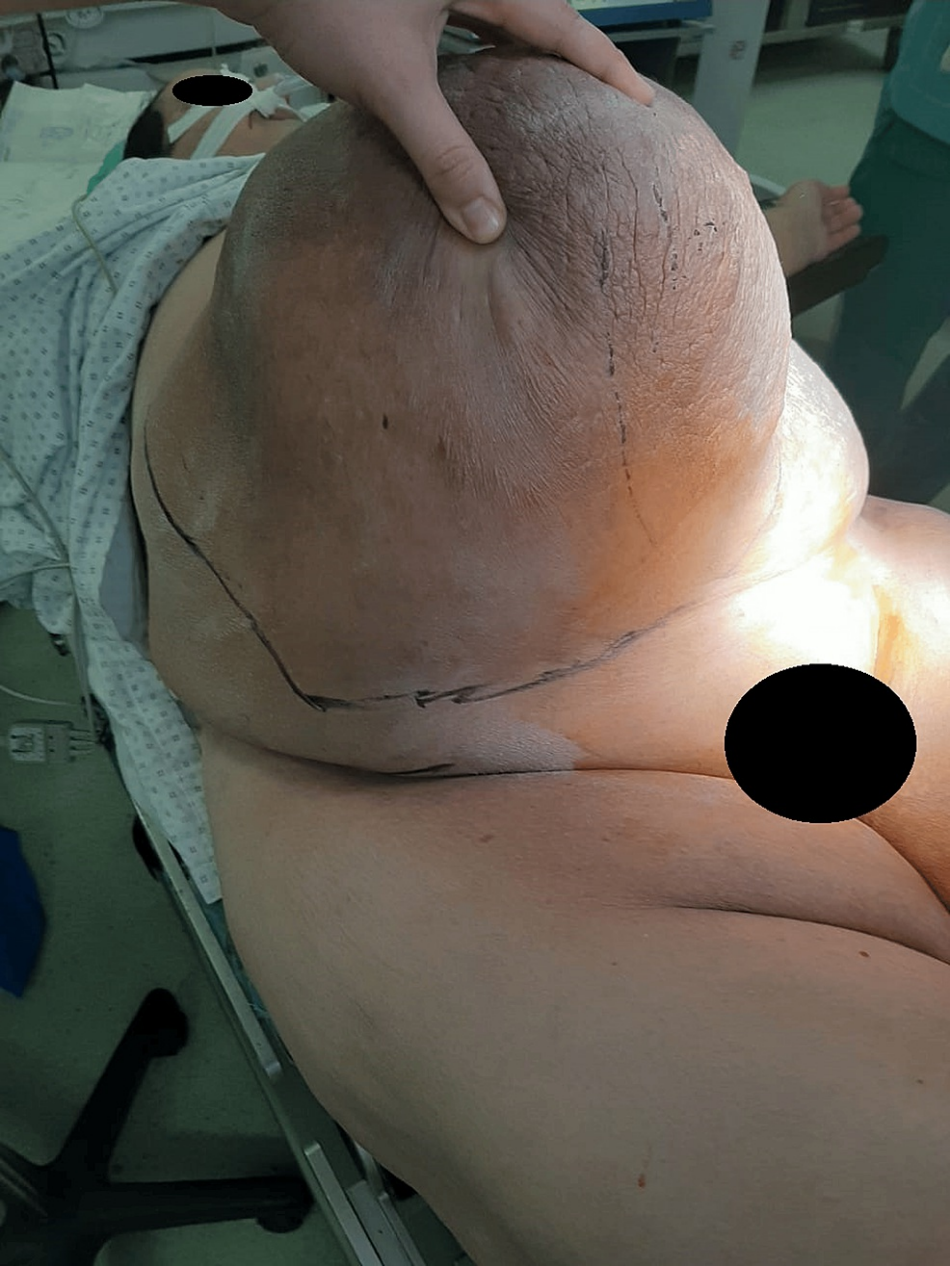
The anterior abdominal mass measuring 25 cm along its major axis.

**Figure 2:**
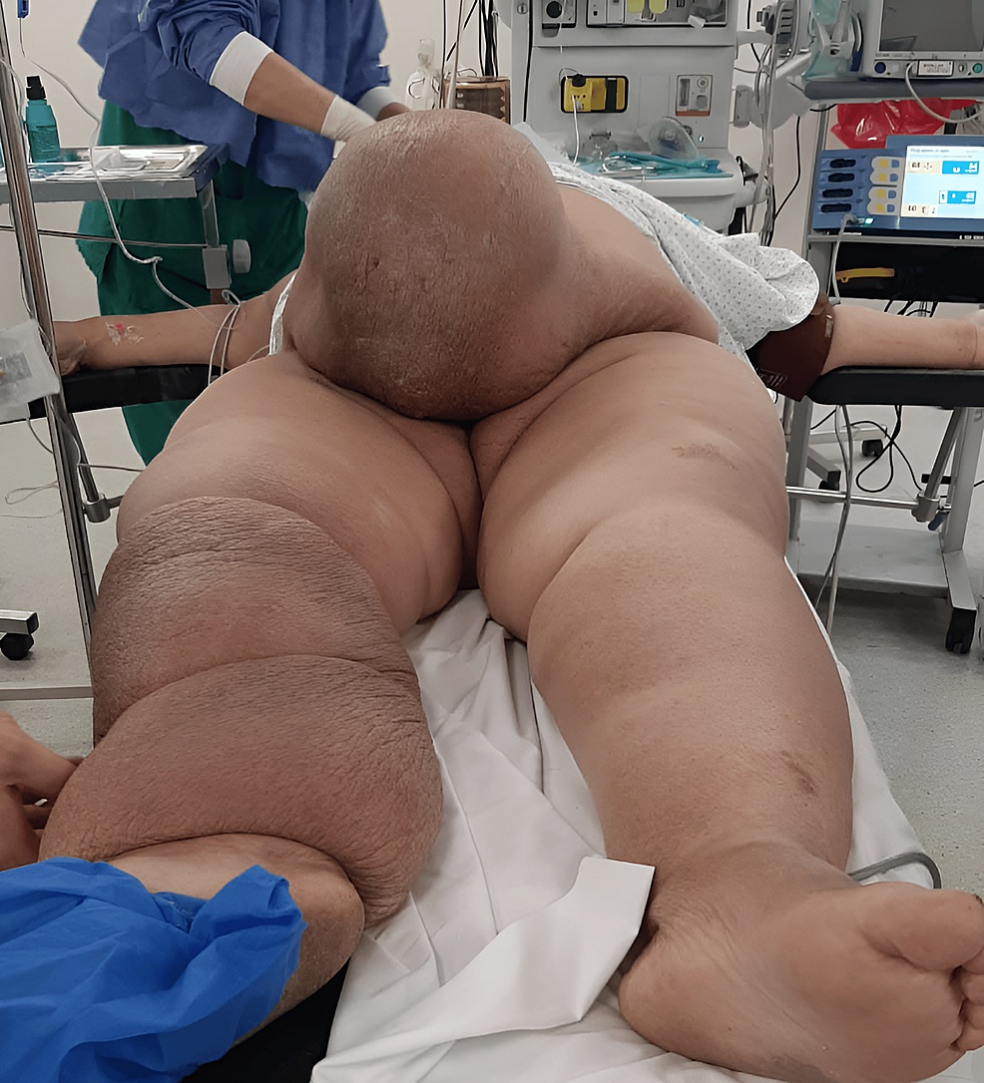
Lymphedema of the right lower limb.

A 55-year-old obese patient, with a body mass index (BMI) of 47, presented with a mass on the anterior abdominal wall without intraabdominal compressive signs, especially digestive, and evolving over seven months with a significant impact on daily quality of life. The mass was painless, measuring 25 cm along its major axis, and occupied the right paraumbilical region (Figure 1). The patient also developed significant lymphedema in the right lower limb five months after the appearance of the mass, associated with superficial inguinal lymphadenopathy on the same side and averaging 2 cm in size (Figure 2). Medical history included a previous cesarean section, anterior abdominal surgery with a longitudinal midline scar, and no noteworthy family history.

**Figure 3:**
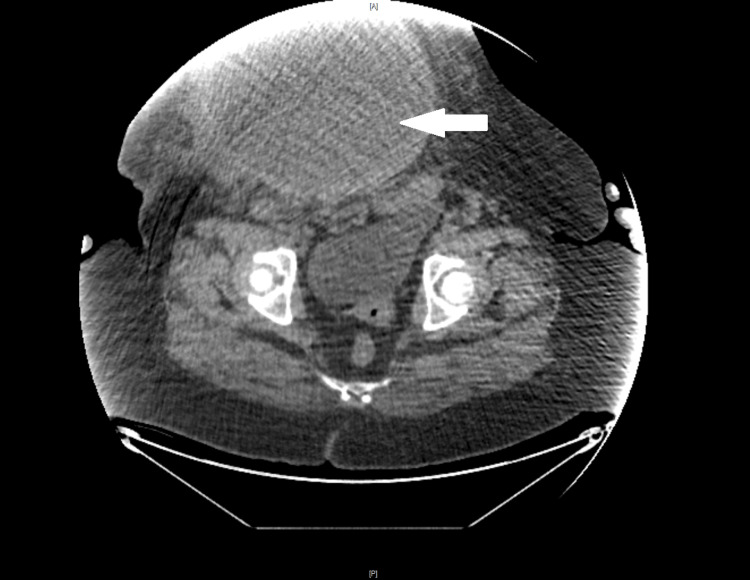
CT image showing the abdominal wall mass (arrow).

Abdominal CT revealed a soft tissue mass in the right paramedian hypogastric region of the anterior abdominal wall, with well-defined tissue density, measuring 159 x 180 mm. This mass did not exhibit clearly identifiable intraperitoneal extension and suggested a desmoid tumor (Figure 3).

**Figure 4:**
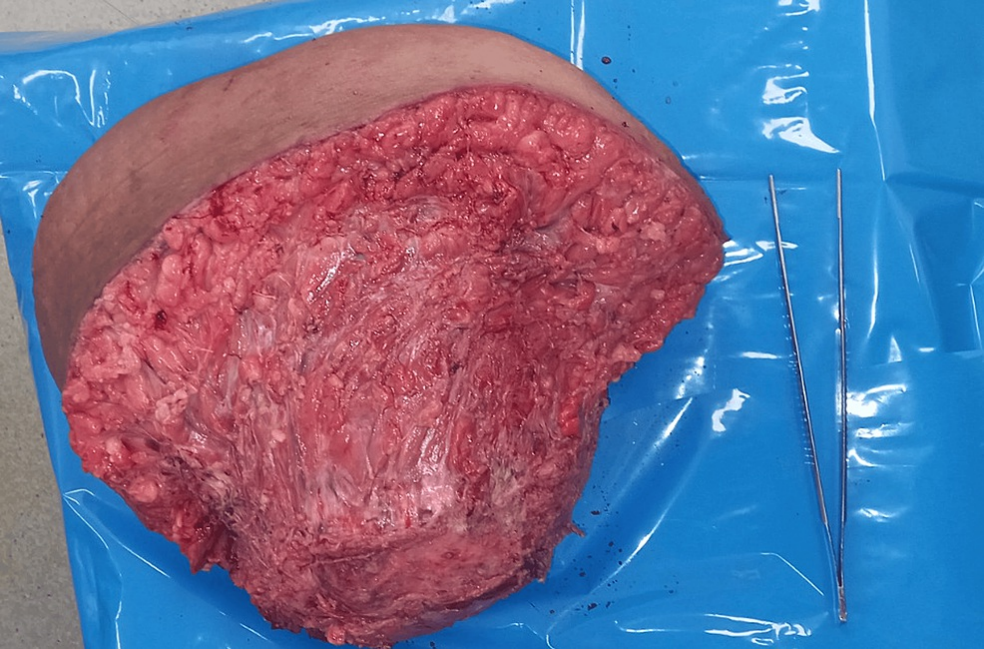
Surgical specimen of the abdominal wall mass.

The surgical approach involved a monobloc resection of the mass with a 5 cm lateral margin, encompassing the right rectus abdominis muscle with its anterior and posterior aponeuroses, the linea alba, and a part of the anterior aponeurosis of the left rectus abdominis muscle (Figure 4).

**Figure 5:**
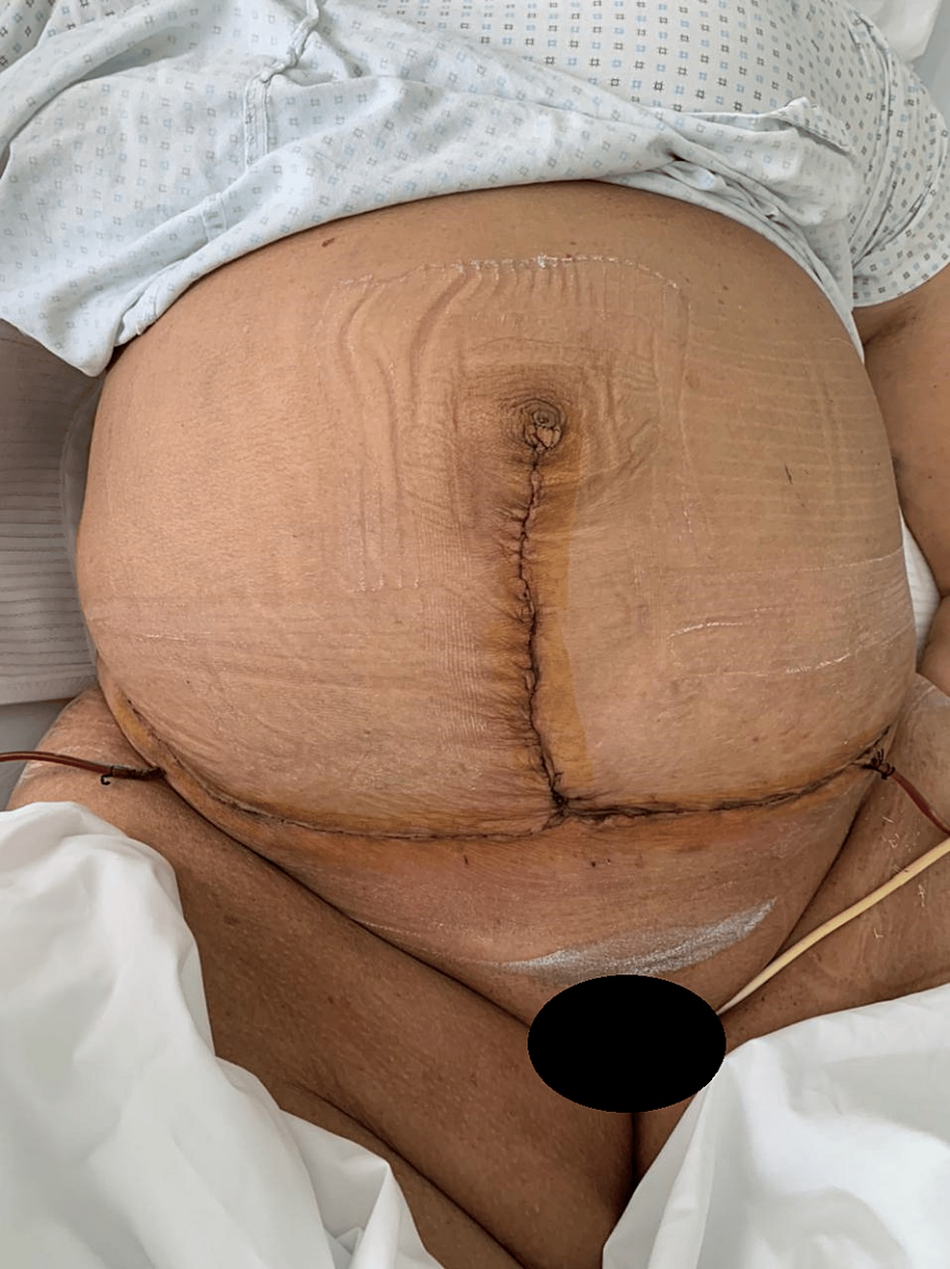
Postoperative scar after skin closure.

Simultaneous reconstruction utilized a biface intraperitoneal synthetic mesh sutured to the edges of the parietal resection, followed by the placement of two subcutaneous aspirative Redon drains (Figure 5).

Postoperative recovery was marked by the resolution of lymphedema in the right lower limb, with a hospitalization duration of 15 days. The patient underwent a rehabilitation program and used compressive bandaging, which contributed significantly to her recovery. She regained full function of her leg and lower limb. Histological and immunohistochemical findings were sufficient to support the diagnosis of a desmoid tumor. However, *CTNNB1* and *APC* mutations were not tested in our case.

The surgical section was performed laterally, maintaining a 5 cm margin from the lesion. After 18 months of postoperative follow-up, the patient showed no signs of recurrence, with acceptable parietal function.

## Discussion

Desmoid-type fibromatosis constitutes a rare clonal proliferation characterized by the aberrant growth of fibroblasts and myofibroblasts. In the comprehensive classification presented by the World Health Organization (WHO) in 2002 regarding the pathology and genetics of soft tissue tumors, this particular entity was categorized as an intermediate or locally aggressive tumor [3]. The classification further stratifies desmoid-type fibromatosis into superficial and deep types, the latter encompassing abdominal, extra-abdominal, and mesenteric manifestations. Distinguished by its locally infiltrative nature and propensity for collagen formation, this tumor exhibits the potential for recurrent occurrences while remaining devoid of metastatic dissemination [4]. Desmoid tumor, recognized as aggressive fibromatosis, represents an infrequent neoplastic entity. Its inaugural documentation traces back to MacFarlane in 1832, with the nomenclature "desmoid" being ascribed by Muller in 1838, derived from the Greek term "desmos", signifying a band of tendon [5]. The reported incidence of desmoid tumors is notably low, ranging from two to five cases per million in the population [6].

The predominant anatomical site for desmoid tumors is the anterior abdominal wall, constituting 50% of reported occurrences. Additional locations include the shoulder girdle, chest wall, and thigh. This neoplasm predominantly affects individuals in their early 30s, with a median age range spanning from 15 to 60 years, and displays a notable twofold to threefold female preponderance [7].

Clinical manifestation commonly involves the presentation of a painless plaque or firm lump, often accompanied by local pressure symptoms. Severe clinical complications may arise when these lesions impinge upon vital vessels, nerves, or abdominal organs, leading to issues such as extremity ischemia, bowel fistulization, perforation, bleeding, and lymphedema [8].

Lymphedema denotes the abnormal accumulation of interstitial fluid resulting in persistent swelling, most commonly observed in extremities. The pathogenesis in this context involves the desmoid tumor impinging upon the lymphatic system, thereby disrupting the normal flow of lymph. The compression or obstruction of lymphatic vessels impedes the drainage of lymph fluid, culminating in a cascade of symptoms and complications [9].

Manifestations associated with desmoid tumor-induced lymphedema encompass localized swelling, compromised immune function due to the perturbation of lymphatic transport of immune cells, inflammatory responses secondary to fluid accumulation, and, in severe cases, diminished mobility and flexibility within the affected limb [9].

Diagnostic imaging modalities for desmoid tumors encompass ultrasonography, MRI, and CT. Ultrasonography is particularly applicable for palpable lesions in various anatomical sites, revealing variable hyperechogenic areas within the tumor and exhibiting well to poorly-defined margins. MRI portrays a diverse signal intensity, resembling muscle on T1-weighted images and displaying intermediate intensity on T2-weighted images, reflecting the heterogeneous distribution of collagen and tumor cellularity. CT scans are also effective in assessing desmoid tumors, typically depicting lower density with homogeneous attenuation and mild enhancement in comparison to muscle tissue [10]. In our case, the data from the CT scan, as well as the reported clinical findings, were sufficient to establish the diagnosis and indicate surgery after a preoperative assessment.

From the literature, it is evident that no single method of treatment is effective. The high recurrence rates after surgical excision may reflect the diffuse infiltrative growth pattern of desmoid tumors. The surgical margins may be difficult to assess, both macroscopically and microscopically. Several studies indicate that wide surgical margins are associated with a lower recurrence rate [8].

The principal therapeutic approach for addressing intraabdominal desmoid tumors is surgical excision. No consensus has been established regarding the surgical technique for closing abdominal defects. Surgeons can choose between using synthetic parietal prostheses or employing free musculocutaneous flaps and aponeurotic approximation techniques [11].

In our case, the surgical approach involved a monobloc resection of the mass with a 5 cm lateral margin and a wall reconstruction using a biface intraperitoneal synthetic mesh sutured to the edges of the parietal resection. Radical tumor resection with free margins has typically been recommended as the first-line therapy. This resection provides a good local control rate of approximately 80% at five years [3]. In instances where positive margins are observed postoperatively, adjunctive interventions such as radiotherapy, chemotherapy, or a combination thereof have been employed, yielding variable degrees of success. The recurrence rate of desmoid tumors subsequent to surgical removal spans a range from nearly one-fifth to more than half [12].

The therapeutic approach to desmoid tumors depends on several factors, including location, patient condition, complications, and the tumor's progression. Therapeutic options vary, ranging from surgery and chemo-radiotherapy to simple surveillance. In our patient's case, there were indications of aesthetic concerns and compressive complications, such as lymphedema, which necessitated immediate surgical resection. The resection of this mass involved the use of prosthetic material, specifically a biface mesh, for abdominal reconstruction.

## Conclusions

Giant desmoid tumors are aggressive fibromatoses characterized by their infiltrative nature and high recurrence potential. As was the case with our patient, the diagnosis is frequently established when CT is combined with clinical evaluation. When problems like lymphedema are present, surgery shouldn't be delayed. To lower the chance of recurrence, a resection with sufficient margins must be performed during surgery. Following resection, the abdominal wall reconstruction may be addressed with a prosthetic parietoplasty. 
